# Protective Effects of the Hydroethanolic Extract of *Fridericia chica* on Undifferentiated Human Neuroblastoma Cells Exposed to α-Zearalenol (α-ZEL) and β-Zearalenol (β-ZEL)

**DOI:** 10.3390/toxins13110748

**Published:** 2021-10-22

**Authors:** Neda Alvarez-Ortega, Karina Caballero-Gallardo, María Taboada-Alquerque, Jackeline Franco, Elena E. Stashenko, Cristina Juan, Ana Juan-García, Jesus Olivero-Verbel

**Affiliations:** 1 Environmental and Computational Chemistry Group, School of Pharmaceutical Sciences, Zaragocilla Campus, University of Cartagena, Cartagena 130014, Colombia; nalvarezo@unicartagena.edu.co (N.A.-O.); kcaballerog@unicartagena.edu.co (K.C.-G.); mtaboadaa@unicartagena.edu.co (M.T.-A.); 2 Functional Toxicology Group, School of Pharmaceutical Sciences, Zaragocilla Campus, University of Cartagena, Cartagena 130014, Colombia; 3 Metabolite Profiling Facility, Bindley Bioscience Center, Purdue University, 1203 W State St., West Lafayette, IN 47907, USA; francoj@purdue.edu; 4 Center for Chromatography and Mass Spectrometry CROM-MASS, Research Center for Biomolecules CIBIMOL, School of Chemistry, Universidad Industrial de Santander, Bucaramanga 680006, Colombia; elena@tucan.uis.edu.co; 5 Facultat de Farmàcia—Avda, Universitat de València, Vicent Andrés Estellés, s/n, 46100 València, Spain; crisjua3@uv.es

**Keywords:** cytotoxicity, mycotoxins, *Fridericia chica*, protection, extracts

## Abstract

*Fridericia chica* (Bignoniaceae) is a traditional medicinal plant. The aim of this research was to determine the protective effects of the hydroethanolic extract from the *F. chica* leaves (HEFc) against the cytotoxicity of zearalenone (α-ZEL) and β-ZEL on SH-SY5Y cells. Free radical scavenging activity of HEFc was evaluated using the DPPH method. The cytotoxicity of both zearalenone metabolites and HEFc was examined using MTT test, as was the cytoprotective effects of the HEFc on cells treated with these mycotoxins. The chemical composition of HEFc was determined using UPLC-QTOF-MS/MS. HEFc elicited good DPPH radical scavenging activity following a concentration-dependent relationship. Cells exposed to α-ZEL exhibited a viability ˂50% after 48 h of treatment (25 and 50 µM), while those exposed to β-ZEL showed viability ˂50% (100 µM) and ˂25% (25-100 µM) after 24 and 48 h of exposure, respectively. HEFc showed a significant increase in cell viability after exposure to α-ZEL (25 and 50 µM) and β-ZEL (6–100 µM) (*p* < 0.05). UPLC-QTOF-MS/MS analyses allowed the identification of 10 phytochemical components in the HEFc. In short, the hydroethanolic extract of *F. chica* grown in Colombian Caribbean can protect against the effects of mycotoxins and it is a valuable source of compounds with antioxidant properties.

## 1. Introduction

Colombia is one of the countries in the world that is considered to be mega-diverse due to its various ecosystems [1]. Bioprospecting has always been a central activity in human development; it is defined as the evaluation of biological material in order to search for new valuable products, and involves the application of advanced technologies for the development of new pharmaceutical, agrochemical products, cosmetics, flavorings, fragrances, industrial enzymes, and other products from biota. The Colombian Caribbean flora offers countless possibilities; an example of this is the *Fridericia chica* (Bonpl.) L.G. Lohmann, Bignoniaceae, which is known as “*limpia diente*” and grows frequently in tropical regions in South America and Africa.

*Fridericia chica* (synonym *Arrabidaea chica*) leaves are usually employed as a red dye, in fact, it has traditionally been used by Sinu artisans of the Colombian Caribbean to make the *vueltiao* hat [2]. The red color obtained from *F. chica* comes from anthocyanidins, a class of phenolic compounds with known antioxidant properties [3], and extracts from the plant have shown pharmacological activities linked to beneficial health effects, including anti-inflammatory [4], antiproliferative [5], wound healing [6], antispasmodic [7], photoprotective [8], and leishmanicidal [9] activities. Many of these properties are attributed to different flavonoid compounds reported in *F. chica*, such as isoscutellarein, 6-hydroxyluteolin, hispidulin, scutellarein, luteolin, apigenin, and hispidulin [10], this last a potential compound for neuroinflammation inhibition [11].

Exposure to environmental toxins is key in the development of neuropathological diseases [12]. Within these pollutants, the mycotoxins are toxic secondary metabolites of fungi and are commonly produced in stored agricultural products [13]. They represent a risk for human and animal health due to their potential to cause disease and death in organisms that are exposed to them [14]. Zearalenone (ZEA) metabolites such as α-zearalenol (α-ZEL) and β-zearalenol (β-ZEL) are produced by the genus *Fusarium* and infect mainly cereals and other plant products [15]. The toxicity of these metabolites is related to their ability to bind to the estrogen receptor and their ability to modify estrogen metabolism [16]. In addition, these metabolites are known oxidative stressors [15,17]. In fact, it has been reported that α-ZEL can induce proliferation in different human cell lines such as MCF7 [18] and granulosa cells [19].

The human neuroblastoma dopaminergic neuronal cell line SH-SY5Y has been used as an in vitro model for numerous neurotoxicity and cytoprotection experiments. The aim of this work was to investigate the potential of a hydroethanolic extract of *F. chica* (HEFc) grown in northern Colombia to mitigate the effects of α-ZEL and β-ZEL in undifferentiated human neuroblastoma, as well as to characterize its constituents.

## 2. Results

### 2.1. DPPH Radical Scavenging

**Figure 1:**
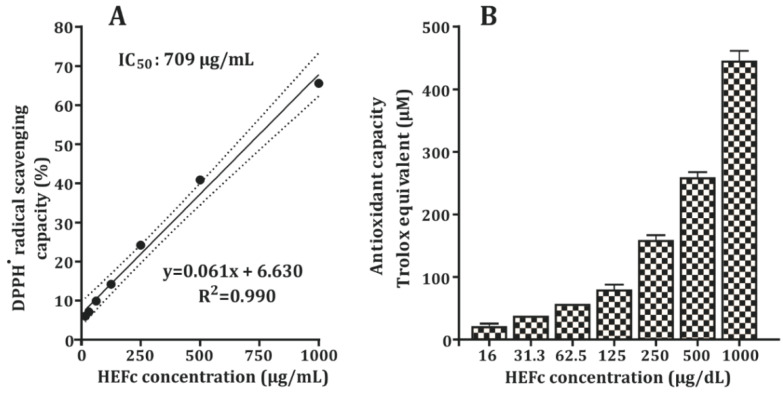
DPPH radical scavenging capacity (**A**) and Antioxidant capacity, Trolox equivalent (µM) (**B**).

The results of the free radical scavenging activity of the HEFc are presented in Figure 1. The HEFc quenched DPPH free radicals in a concentration-dependent manner. The antioxidant assay produced values in the range of 21 ± 4.5–430 ± 27.8 µM Trolox. At 16 µg/mL, HEFc showed a low DPPH inhibition of 6%, and at 1000 µg/mL, it resulted in 65% DPPH inhibition. In addition, the IC_50_ value was found to be 709 µg/mL.

### 2.2. Cytotoxicity of HEFc and Mycotoxins on SH-SY5Y Cells

**Figure 2:**
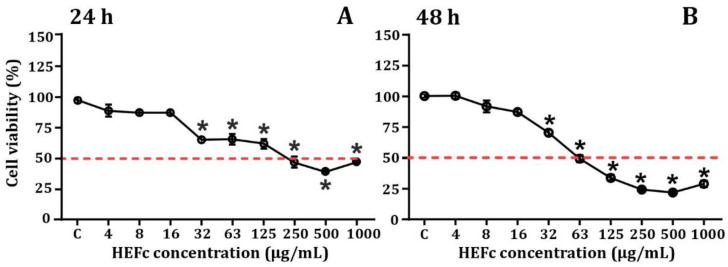
Efect of HEFc on SH5YSY viability. (**A**) Cytotoxicity effects in undifferentiated human neuroblastoma cells exposed to HEFc for 24 h, (**B**) Cytotoxicity effects in undifferentiated human neuroblastoma cells exposed to HEFc for 48 h. * *p* ˂ 0.05, representing a significant difference compared to the control. Data are mean ± SEM (*n* = 3), one-way ANOVA post Sidak’s multiple comparisons test.

The cytotoxic activity of the HEFc over 24 and 48 h in undifferentiated human neuroblastoma cells is presented in Figure 2. The HEFc was found to significantly reduce cell viability in SH-SY5Y cells. It was determined that exposure to a concentration of 16 µg/mL for 24 to 48 h periods did not affect viability (Figure 2). The corresponding IC_50_ values for SH-SY5Y cells at 24 and 48 h were 61.2 µg/mL (45 to 83 µg/mL) and 53.8 µg/mL (24.0 to 116.6 µg/mL), respectively (Table 1).

**Figure 3:**
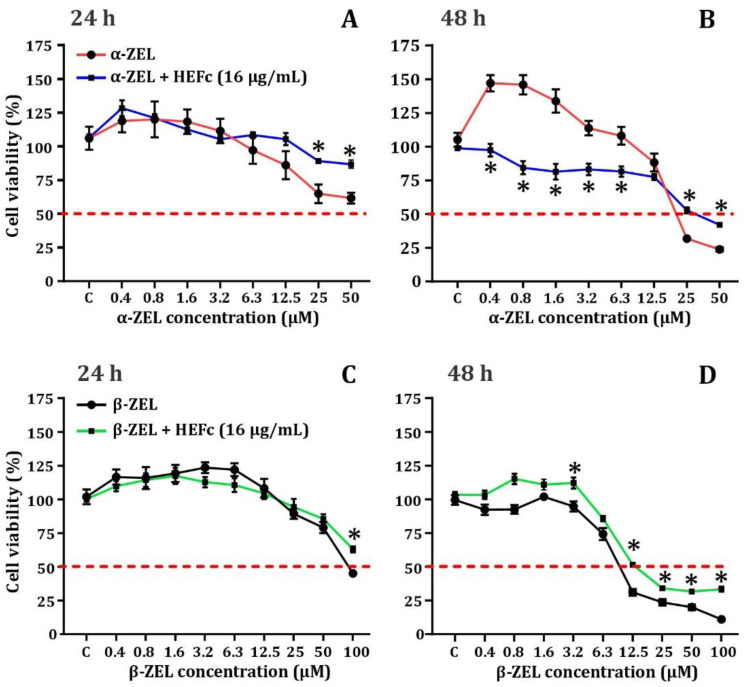
Cell viabillity in SH-SY5Y exposed to α-ZEL/α-ZEL/HEFc (**A**,**B**) or β-ZEL/β-ZEL/HEFc (**C**,**D**) over 24 (**A**,**C**) and 48 h (**B**,**D**). * *p* ˂ 0.05, significant difference compared to the correspondiong metabolite tested alone. Data are mean ± SEM (*n* = 3). Multiple t-tests.

The cytotoxicity of α-ZEL and β-ZEL in undifferentiated human neuroblastoma cells are displayed in Figure 3. Cell proliferation increased at low concentrations (0.4, 0.8 and 1.6 µM), after 24 and 48 h of exposure; β-ZEL also caused a similar behavior, but only during the first 24 h and the IC_50_ could not be calculated after 24 h of treatment; while at 48 h of treatment, the IC_50_ values were 17.9 µM (CI_95_: 10.4 to 32.4 µM) for α-ZEL and 10.5 µM (CI_95_: 7.1 to 15.7 µM) for β-ZEL (Table 1).

### 2.3. Cytoprotective Effects of HEFc against Zearalenone Metabolites

The protective effects of HEFc against cytotoxicity in SH-SY5Y cells induced by α-ZEL and β-ZEL after 24 and 48 h exposures, are shown in Figure 3. The results showed that when SH-SY5Y cells were simultaneously treated with α-ZEL (25 and 50 μM) and HEFc (16 µg/mL), the extract exhibited significant cytoprotection (24–25%, *p* < 0.05) after the first 24 h of incubation. HEFc (16 µg/mL) significantly increased in cell proliferation in samples with low concentrations of α-ZEL (0.4, 0.8, 1.6, 3.2, and 6.3 μM, *p* < 0.05) after treatment for 48 h. At the highest α-ZEL concentrations tested (25–50 µM), simultaneous co-exposure with the extract was able to significantly increase cell viability (*p* < 0.05) by 18–21%.

The treatment with HEFc (16 µg/mL) in undifferentiated human neuroblastoma cells exposed for 24 h with β-ZEL only displayed some protection in viability (~20%) at the highest tested mycotoxin concentration (100 µM) tested. After prolonged exposure (48 h), HEFc was able to significantly recover cell viability at an extent similar to that observed after 24 h (~20%); however, the concentration range displaying the effect was greater (12.5–100 μM) (Figure 3).

### 2.4. UPLC-QTOF-MS/MS Analysis

**Figure 4:**
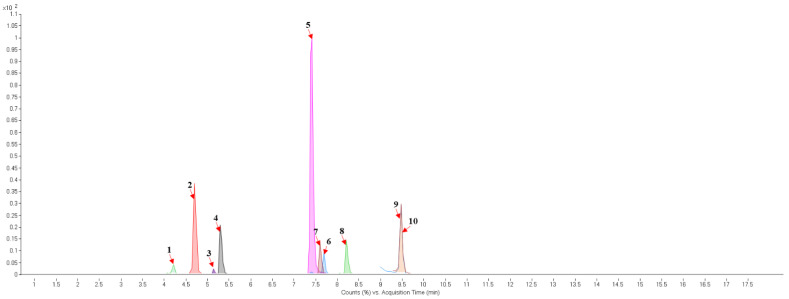
Chromatograms of *Fridericia chica*. Extract chromatogram Rt: 3–11 min. (extracted ion chromatogram, EIC), obtained by UPLC-MS/MS in positive ion mode. Numbers correspond to (1) vicenin-2; (2) 6-hydroxyluteolin 7-rhamnoside; (3) scutellarein-O-glucuronide; (4) nepetin; (5) pectolinarigenin; (6) hispidulin; (7) apigenin; (8) 5-O-methylscutellarein; (9) thevetiaflavone and (10) acacetin.

The results of ultra-performance liquid chromatography/quadrupole time-of-flight mass spectrometry (UPLC-QTOF-MS/MS) analysis of the HEFc are shown in Figure 4 and Table 2. The identified compounds belong to various classes, including flavone glycosides (Vicenin-2, 6-hydroxyluteolin 7-rhamnoside and Scutellarein-O-glucuronide) and flavones (Nepetin, Pectolinarigenin, Hispidulin, Apigenin, 4′,6,7-trihydroxy-5-methoxyflavone, Thevetiaflavone and Acacetin), among others. The extracted ion chromatogram (Rt: 3–11 min, EIC) for the HEFc obtained by UPLC-QTOF-MS/MS in positive ion mode, the exact mass characteristics for positive ions of identified chemicals, and fragment spectrum results (MS/MS) are presented in Supplementary Material.

## 3. Discussion

This study evaluated the free radical scavenging activity of a hydroethanolic extract of *F. chica* (HEFc) obtained from fallen leaves collected from trees grown in the Colombian Caribbean, the protective effects of the extract on zearalenone metabolites-induced citotoxicity in SH-SY5Y cells, and its chemical composition.

*F. chica* is a plant widely known for its anti-inflammatory [4,5], antibacterial [20,21] healing actions [6], and antioxidant properties [22,23]. However scientific studies of its properties from fallen leaves of plants cultivated in Colombia are scarce. The DPPH scavenging activity was dependent concentration of HEFc. The IC_50_ of the extract was 709 µg/mL, a value much than that reported in extracts obtained from leaves of the same species from Brazil (IC_50_ of 13.5 µg/mL) [10] or Argentina (57.84 µg/mL) [23]. These differences may result from distinct climatic conditions or soil properties of the sites where they have been collected, but this is unclear, as this is the first study that reports the DPPH antiradical activity of HEFc in the Colombian Caribbean. The antioxidant properties of this plant can be explained by the presence of flavonoids, alkaloids, and phenolic compounds found in phytochemical screening also reported by other authors [24,25].

Although some biological properties of *F. chica* have been previously reported [23,25]; the present research is the first to evaluate the response on the viability of undifferentiated human neuroblastoma cells exposed to HEFc from the Colombian Caribbean. Interestingly, cell proliferation was not observed under any of the concentrations evaluated. The IC_50_ found here after 24 and 48 h treatment (61.2 and 53.8 µg/mL), were moderately higher than the IC_50_ values (<30 µg/mL) suggested as a criteria to extract promising agents for anticancer drug development [26]. However, a similar extract has shown good activities in other cancer cell lines, such as HL60 (IC_50_, 26.9 µg/mL), and Jurkat cells (IC_50_, 27.9 µg/mL) [5]. Notably, the extract has been reported to show growth stimulation in several cell lines, including fibroblasts [6], NIH-3T3 cells [27], and CHO-K1 cells [20].

Zearalenone metabolites at high concentrations produce an increase in cell death after the first 24 h of treatment, finally reaching values below the IC_50_ after 48 h of exposure. These results are similar to those obtained by other authors [16,28]. However, there are differences with respect to the concentrations required to reach IC_50_ values after 48 h of exposure. In the present study, it was found that a concentration of 17.9 µM (α-ZEL) and 10.5 µM (β-ZEL) was required to reach the IC_50_ values in SH-SY5Y cells, while other types of cell lines appear less sensitive (e.g., IC_50_ of 32 and 55 µM for α-ZEL and β-ZEL, respectively in CHO-K1 cells) [28]. Interestingly, α-ZEL induced and increase in cell proliferation at low concentrations. These results are consistent with those reported by other authors [19]. It is known that cell proliferation is an essential event in various physiological processes, such as tissue generation, but also in various pathophysiological events such as cancer formation [29]. In this last case, the evidence suggests ZEA metabolites stimulate cell proliferation and therefore may promote cancer in different cells [30].

The brain is also a target for estrogens and phytoestrogens, such as ZEA or its metabolites, as they are known to cross the blood-brain barrier [31]. Although data are limited and their role of mycotoxins in neurodegerative diseases is not yet understood, a recent study showed these molecules alter the expression of dopaminergic genes in SH5YSY cells [32]. In this work, the HEFc exerted an antiproliferative effect on neuronal cells, suggesting it has propective properties on ZEA metabolites-induced cell proliferation.

The characterization of HEFc showed that some of the tentatively identified compounds has been reported to have potential anti-osteoporotic, anti-inflammatory, antiplatelet, anticonvulsant, and anticancer [33,34]. Flavone compounds such as nepetin, pectolinarigenin, apigenin, taxifolin, hispidulin, thevetiaflavone, and acacetin present in the HEFc have been reported as potential neuroprotective agents for their ability to suppress neuronal apoptosis, oxidative stress and inflammation by attenuating levels of malondialdehyde, lactate dehydrogenase, Bax, caspase-3, TNF-α, IL-1β and IL-6, and increasing levels of Bcl-2 and SOD [35,36,37,38,39,40].

## 4. Conclusions

The present study demonstrates that hydroethanolic extract from the *F. chica* leaves exerts significant protective effects against mycotoxin-induced cytotoxicity in undifferen-tiated human neuroblastoma cells. This may be due to the presence of compounds with antioxidant properties present in the extract. These results may contribute to new approaches for treatment against the effects of mycotoxins.

## 5. Materials and Methods

### 5.1. Plant Material and Extraction

The fallen leaves of *F. chica* (Bonpl.) L.G. Lohmann, Bignoniaceae were collected between January and December 2018 in the municipality of Sincelejo (9°14′20″ N-75°25′17″ W), department of Sucre (Sincelejo, Colombia). The specimens were identified by Pedro Alvarez Perez in the Herbarium of the University of Sucre (Sincelejo, Colombia), and a voucher specimen was deposited and registered in this herbarium (004537). Leaves of *F. chica* were dried at room temperature. Subsequently, dried leaves were cut and powder (30 g) was mixed with (1:10, *w*/*v*) with 70% ethanol in water for 4 h at 200 rpm using an automated Shaker. The extract was filtered and the solvent evaporated in an oven at 70 °C under a hood. The extract was a black reddish solid, and was stored at −20 °C until analysis.

### 5.2. DPPH Radical-Scavenging Method

DPPH radical scavenging assay was determined using the kit from Bioquochem (Llanera, Asturias, Spain). Initially, a stock solution at 100,000 µg/mL of the hydroethanolic extract from the HEFc with MilliQ water was prepared. All extract samples were tested in duplicate at seven different concentrations (1000, 500, 250, 125, 62.5, 32.5, and 16 µg/mL), then, sample extracts (20 μL) were reacted with 200 μL of the DPPH solution in the dark at room temperature. The absorbance at 517 nm was performed in the Varioskan™ LUX Multimode Microplate Reader (Thermo Fisher Scientific, Inc., Waltham, MA, USA). The percentage of inhibition of the radical DPPH•+ for each standard point was obtained with the following formula:

% Inhibition = [1 − (Abs Sn/Abs S1)] × 100(1)

where Abs S1 is the DPPH•+ radical absorption without inhibition and Abs Sn is the DPPH•+ radical absorption of the correspondent standard.

A Trolox^R^ standard curve was used to determine TEAC (Trolox Equivalent Antioxidant Capacity) of the tested concentrations of extracts. The calculation was performed using the following formula:

TEAC (µM) = % inhibition − intercept/slope(2)

### 5.3. Cellular Exposure to Mycotoxins and HEFc

SH-SY5Y NB cells (ATCC^®^ CRL-2266™) were maintained following the methods previously reported [41]. The cells were incubated in 96-well plates (2×10^6^ cells/plate) for 24 h. Briefly, the cells were exposed to 100 μL of medium containing different concentrations of α-ZEL (0.4 to 50 μM), β-ZEL (0.4 to 100 μM) and HEFc (4 to 1000 µg/mL) individually for 24 and 48 h. For all cases, the viability was examined using MTT test (Section 5.5), and absorbance was determined using a spectrophotometer at 570 nm (VICTOR x5 Multimode Microplate, PerkinElmer’s, Waltham, MA, USA). Three experiments were carried out with four replicates each. Concentration-effect curves were built to obtain IC_50_ using Prism 6.0 (GraphPad Software Inc.).

### 5.4. Cytoprotective Effects of HEFc against ZEN Metabolites

SH-SY5Y cells incubated in 96-well plates (2 × 10^6^ cells/plate) for 24 h at 37 °C in a 5% CO_2_ atmosphere and these were exposed to two independent treatment combinations. The first consisted of a mixture of HEFc (16 µg/mL) with eigth concentrations of α-ZEL (from 0.4 to 50 µM, 1: 2 dilutions), for the second, the combination of HEFc (16 µg/mL) and nine concentrations of ZEN metabolites (0.4 to 100 μM, 1: 2 dilutions) for 24 and 48 h were tested. The plates were incubated for 24 and 48 h at 37 °C, in a 5% CO_2_ atmosphere and the viability was examined by MTT assay (Section 5.5). Three experiments were carried out with four replicates each.

### 5.5. MTT Assay

Viability was examined using the MTT assay as previously reported [42,43]. Briefly, medium with treatments (HEFc, mycotoxins, and mycotoxins/HEFc), as previously described, was removed and each well received 200 μL of medium with 50 μL of MTT solution (5 mg/mL). After an incubation period of 4 h at 37 °C, the MTT containing media was discarded and 200 μL of DMSO and 25 μL of Sorensen’s solution were added to each well prior to reading optical density at 570 nm, using a VICTOR x5 multimode plate reader (PerkinElmer, Waltham, MA, USA). All experiments were performed in three independent experiments with four replicates for each treatment.

### 5.6. Analysis of the HEFc by UPLC-QTOF-MS/MS

The HEFc was diluted 1/10 in 50% acetonitrile mixed with 0.1% HCOOH. Samples were sonicated for 5 min and then centrifuged at 16,000 *g* (8 min). The supernatants were transferred to auto sampler vials. Ultra-performance liquid chromatography/quadrupole time-of-flight mass spectrometry (UPLC-QTOF-MS/MS) was utilized to separate and characterize the components. Chromatographic separation was performed using an Agilent 1290 UPLC system (Agilent Technologies, Palo Alto, CA, USA) employing a YMC Carotenoid column (3 μm particle size, 2.0 × 150 mm) (YMC America Allenton, PA, USA) with a mobile phase flow rate of 0.3 mL/min, where the mobile phase A and B were 0.1% HCOOH acid in ddH_2_O and acetonitrile, respectively. Starting conditions were 95:5 A: B, held for 1 min, followed by a linear gradient to 5:95 at 12 min, with a hold to 15 min. Column re-equilibration was accomplished by returning to 95:5 A:B at 16 min and holding until 21 min. The mass analysis was acquired with an Agilent 6545 Q-TOF MS (Agilent Technologies, Santa Clara, CA, USA) with ESI capillary voltage +3.5 kV, N_2_ gas temperature 320 °C, drying gas flow rate 8.0 L/min, nebulizer gas pressure 35 psig, fragmentor voltage 135 V, skimmer 65 V, and OCT RF 750 V. Mass spectral data were gathered in profile mode. Mass accuracy was enhanced by infusing Agilent Reference Mass Correction Solution (G1969-85001). The instrument was operated from 100 to 1200 *m/z* at a scan rate of 2 spectra/s. MS data scans were obtained using Agilent MassHunter Acquisition software (v. B.06). MS/MS was achieved in a data-dependent acquisition mode on composite samples. Peak deconvolution and integration was executed using Agilent ProFinder (v. B.06). Peak annotations were carried out with METLIN (metlin.scripps.edu) metabolite databases, with a mass error around 1 ppm. Identifications were supported by MS/MS spectra comparisons.

### 5.7. Statistical Analysis

The data are presented as mean±SEM and analyzed statistically by GraphPad Prism 8.0 (GraphPad Prisma Software, Inc., San Diego, CA, USA). The IC_50_ values of HEFc and ZEN metabolites were analyzed using a non-linear sigmoid curve fit. Statistical comparison was performed using one-way analysis of variance (ANOVA) with Sidak’s multiple comparisons test. In addition, multiple Student *t*-test was conducted to investigate different mean cell viabillity between mixture (extract and metabolite) and metabolite alone. Results were considered significant at *p* < 0.05.

## Figures and Tables

**Table 1:** Medium inhibitory concentration IC_50_ in SH-SY5Y cells exposed to zearalenone metabolite and HEFc over 24 and 48 h periods.

Treatment	IC_50_ (CI_95_) 24 h	IC_50_ (CI_95_) 48 h
HEFc	61.2 µg/mL (45–83)	53.8 µg/mL (24.0–116.6)
α-ZEL	>50 µM	17.9 µM (10.4–32.4)
β-ZEL	>100 µM	10.5 µM (7.1–15.7)

**Table 2:** Top results of UPLC-QTOF-MS/MS analysis.

No. Figure 4	RT (min)	Tentative Annotation	Structure	Formula	Ion	Experimental Mass	Calculated Mass	Δ ppm
1	4.204	Vicenin-2	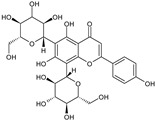	C_27_H_30_O_15_	[M+H]^+^	594.1571	594.15847	−2.30
2	4.732	6-hydroxyluteolin 7-rhamnoside	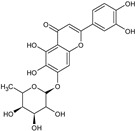	C_21_H_20_O_11_	[M+H]^+^	448.10071	448.10056	0.33
3	5.158	Scutellarein-O-glucuronide	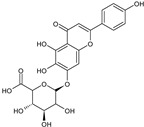	C_21_H_18_O_12_	[M+H]^+^	462.07918	462.07983	−1.40
4	5.355	Nepetin	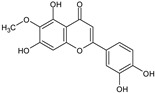	C_16_H_12_O_7_	[M+H]^+^	316.05831	316.0583	0.03
5	7.443	Pectolinarigenin	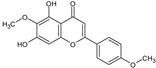	C_17_H_14_O_6_	[M+H]^+^	314.07919	314.07904	0.47
6	7.578	Hispidulin	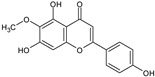	C_16_H_12_O_6_	[M+H]^+^	300.06317	300.06339	−0.73
7	7.631	Apigenin	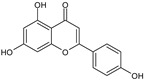	C_15_H_10_O_5_	[M+H]^+^	270.05259	270.05282	−0.85
8	8.254	5-O-methylscutellarein	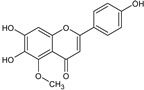	C_16_H_12_O_6_	[M+H]^+^	300.06367	300.06339	0.93
9	9.331	Thevetiaflavone	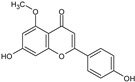	C_16_H_12_O_5_	[M+H]^+^	284.0685	284.06847	0.10
10	9.572	Acacetin	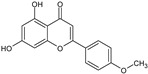	C_16_H_12_O_5_	[M+H]^+^	284.06872	284.06847	0.88

## Supplementary Materials

The following are available online at https://www.mdpi.com/article/10.3390/toxins13110748/s1, Table S1: Exact mass characteristic positive ions of compounds identified by UPLC-QTOF-MS/MS in *Fridericia chica* extract, Figure S1: Compound fragment spectrum results (MS/MS).

## Abbreviations

CI_95_	95% confidence interval
IC_50_	inhibitory concentration
α-ZEL	α-zearalenol
β-ZEL	β-zearalenol
HEFc	hydroethanolic extract from *F. chica* leaves
SEM	standard error of mean
ZEN	zearalenone
