# Supplementary material for: Protective Effects of the Hydroethanolic Extract of Fridericia chica on Undifferentiated Human Neuroblastoma Cells Exposed to α-Zearalenol (α-ZEL) and β-Zearalenol (β-ZEL)
Source: Toxins (Basel). 2021 Oct 22;13(11):748. doi: 10.3390/toxins13110748 (PMC8618744; doi:10.3390/toxins13110748)
Supplement: Supplementary file 1 [file toxins-13-00748-s001.zip › toxins-1419709 supplementary.pdf]

Supplementary Materials: Protective Effects of the Hydroethanolic Extract of *Fridericia chica* on Undifferentiated Human Neuroblastoma Cells Exposed to α-Zearalenol (α-ZEL) and β-Zearalenol (β-ZEL)

Neda Alvarez-Ortega, Karina Caballero-Gallardo, María Taboada-Alquerque, Jackeline Franco, Elena E. Stashenko, Cristina Juan, Ana Juan-García and Jesus Olivero-Verbel

Contents

1. Supplementary Table S1. Exact mass characteristic positive ions of compounds identified by UPLC-QTOF-MS/MS in *Fridericia chica* extract.

2. Compound fragment spectrum results (MS/MS).

Table S1. Exact mass characteristic positive ions of compounds identified by UPLC-QTOF-MS/MS in *Fridericia chica* extract.

No. Fig. S1	Fragment ions			Identification criteria	References
	Fragment type	Fragment formula	m/z		
1	$[M+H]^+$<br>$[M+H-H_2O]^+$<br>$[M+H-C_4H_8O_4]^+$<br>$[M+H-H_2O-C_4H_8O_4]^+$<br>$[M+H-C_4H_8O_4-C_5H_{10}O_5]^+$	$[C_{27}H_{31}O_{15}]^+$<br>$[C_{27}H_{29}O_{14}]^+$<br>$[C_{23}H_{23}O_{11}]^+$<br>$[C_{23}H_{21}O_{10}]^+$<br>$[C_{18}H_{13}O_6]^+$	595.16445<br>577.15256<br>475.12146<br>457.11136<br>325.06927	a	[1, 2]
2	$[M+H]^+$<br>$[M+H-H_2O]^+$<br>$[M+H-C_4H_8O_4]^+$<br>$[M+H-C_5H_{10}O_5]^+$	$[C_{21}H_{21}O_{11}]^+$<br>$[C_{21}H_{19}O_{10}]^+$<br>$[C_{17}H_{13}O_7]^+$<br>$[C_{16}H_{11}O_6]^+$	449.10791<br>431.09599<br>329.06487<br>299.05412	a	[3]
3	$[M+H]^+$<br>$[M+H-C_6H_8O_6]^+$	$[C_{21}H_{19}O_{12}]^+$<br>$[C_{15}H_{11}O_6]^+$	463.08709<br>287.05390	a	[4]
4	$[M+H]^+$<br>$[M+H-CH_3]^+$<br>$[M+H-CH_3-C_8H_6O_2]^+$<br>$[M+H-CH_3-CO-C_8H_6O_2]^+$	$[C_{16}H_{13}O_7]^+$<br>$[C_{15}H_{10}O_7]^+$<br>$[C_7H_4O_5]^+$<br>$[C_6H_4O_4]^+$	317.06545<br>302.04162<br>168.00428<br>140.00976	b	[3, 5]
5	$[M+H]^+$<br>$[M+H-CH_3]^+$<br>$[M+H-2CH_3-CO]^+$<br>$[M+H-2CH_3-C_8H_5O]^+$	$[C_{17}H_{15}O_6]^+$<br>$[C_{16}H_{12}O_6]^+$<br>$[C_{14}H_9O_5]^+$<br>$[C_7H_4O_5]^+$	315.08649<br>300.06299<br>257.04335<br>168.00477	a	[6]
6	$[M+H]^+$<br>$[M+H-CH_3]^+$<br>$[M+H-CH_3-C_8H_6O]^+$	$[C_{16}H_{13}O_6]^+$<br>$[C_{15}H_{10}O_6]^+$<br>$[C_7H_4O_5]^+$	301.07016<br>286.04677<br>168.00477	b	[7, 5]

**7**	$[M+H]^+$<br>$[M+H-C_8H_6O]^+$	$[C_{15}H_{11}O_5]^+$<br>$[C_7H_5O_4]^+$	271.06018<br>153.0115	b	[8, MassBank code: CCMSLIB00000848378)
**8**	$[M+H]^+$<br>$[M+H-CH_3]^+$<br>$[M+H-CH_3-CO]^+$<br>$[M+H-CH_3-C_8H_5O]^+$	$[C_{16}H_{13}O_6]^+$<br>$[C_{15}H_{10}O_6]^+$<br>$[C_{14}H_{10}O_5]^+$<br>$[C_7H_5O_5]^+$	301.07061<br>286.04669<br>258.05211<br>169.01260	a	[7]
**9**	$[M+H]^+$<br>$[M+H-CH_3]^+$<br>$[M+H-CH_3-CO]^+$	$[C_{16}H_{13}O_5]^+$<br>$[C_{15}H_{10}O_5]^+$<br>$[C_{14}H_{10}O_4]^+$	285.07564<br>270.05204<br>242.05713	a	[1]
**10**	$[M+H]^+$<br>$[M+H-CH_3]^+$<br>$[M+H-CH_3-CO]^+$<br>$[M+H-CH_3-C_8H_5O]^+$	$[C_{16}H_{13}O_5]^+$<br>$[C_{15}H_{10}O_5]^+$<br>$[C_{14}H_{10}O_4]^+$<br>$[C_7H_5O_4]^+$	285.07554<br>270.05188<br>242.05709<br>153.01768	b	[3, MassBank code: BML00818)

[a] Tentative identification based on m/z positive mode, reported in the literature for *Fridericia chica* or other species of the Bignoniaceae family.

[b] Tentative identification based on the fragmentation pattern study (ESI-QTOF) and data reported in scientific articles

2. Figure S1: Compound fragment spectrum results (MS/MS).

Compound 1

Retention time: 4.204

m/z [M+H]: 595.16445

Formula: $C_{27}H_{30}O_{15}$

Tentative annotation: Vicenin-2

Compound class: Flavone glycosides

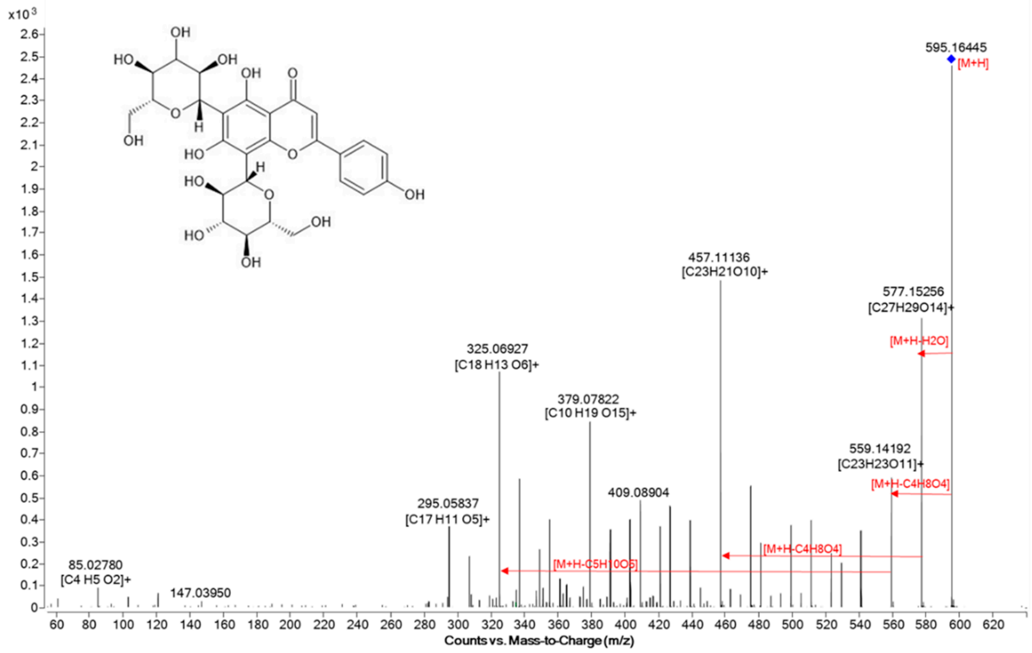

Compound 2

Retention time: 4.732

m/z [M+H]: 449.10791

Formula: $C_{21}H_{20}O_{11}$

Tentative annotation: 6-hydroxyluteolin 7-rhamnoside

Compound class: Flavone glycosides

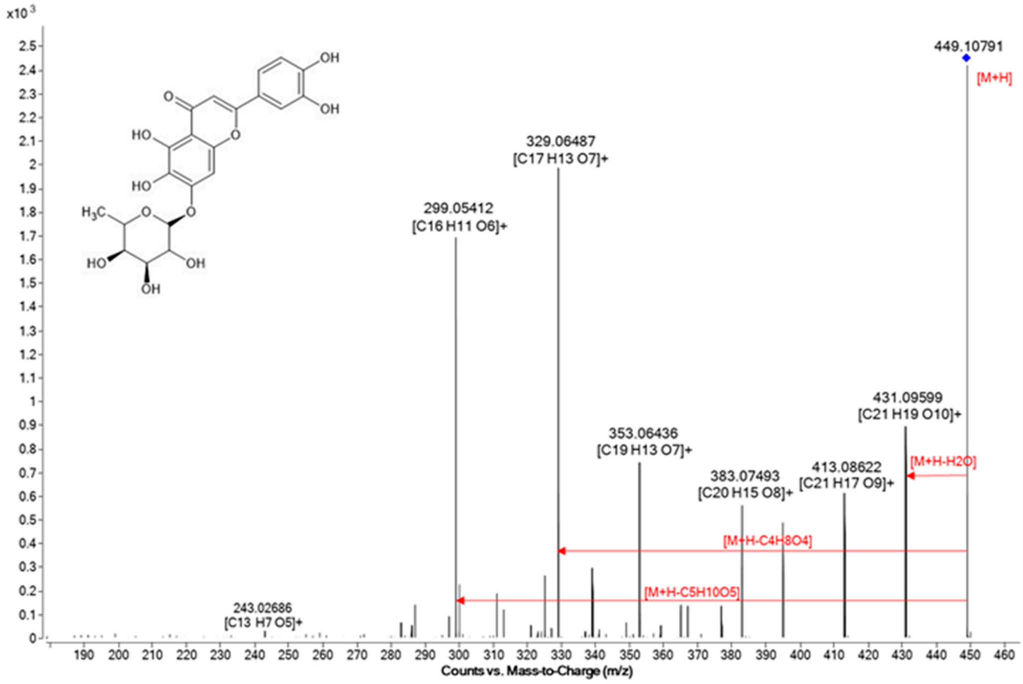

Compound 3
Retention time: 5.158
m/z [M+H]: 463.08709
Formula: $C_{21}H_{18}O_{12}$
Tentative annotation: Scutellarein-O-glucuronide
Compound class: Flavone glycosides

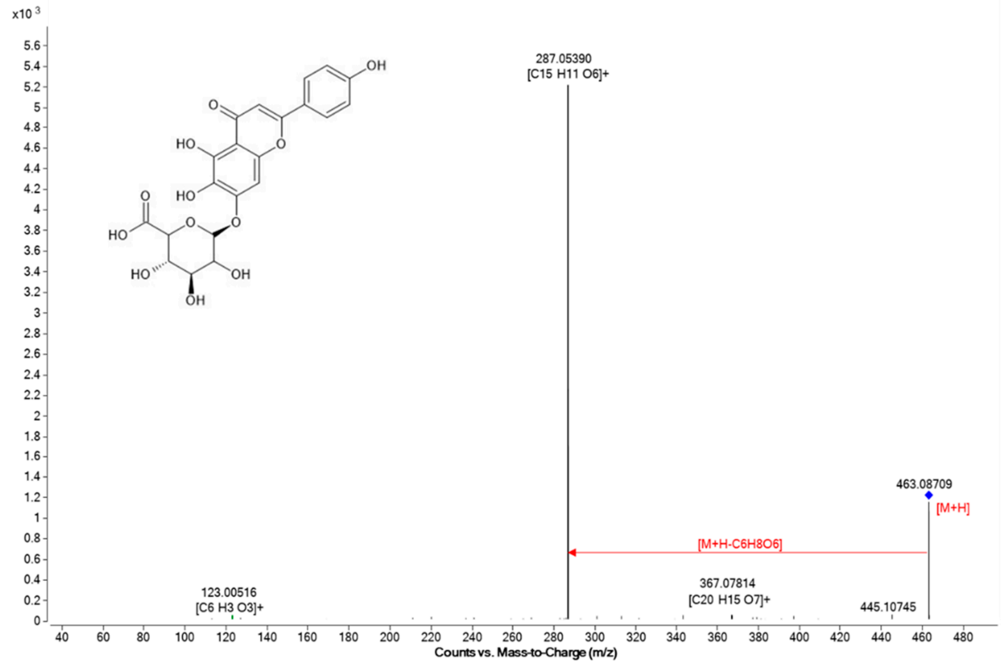

Compound 4
Retention time: 5.355
m/z [M+H]: 317.06545
Formula: $C_{16}H_{12}O_7$
Tentative annotation: Nepetin (6-Methoxyluteolin)
Compound class: Flavone

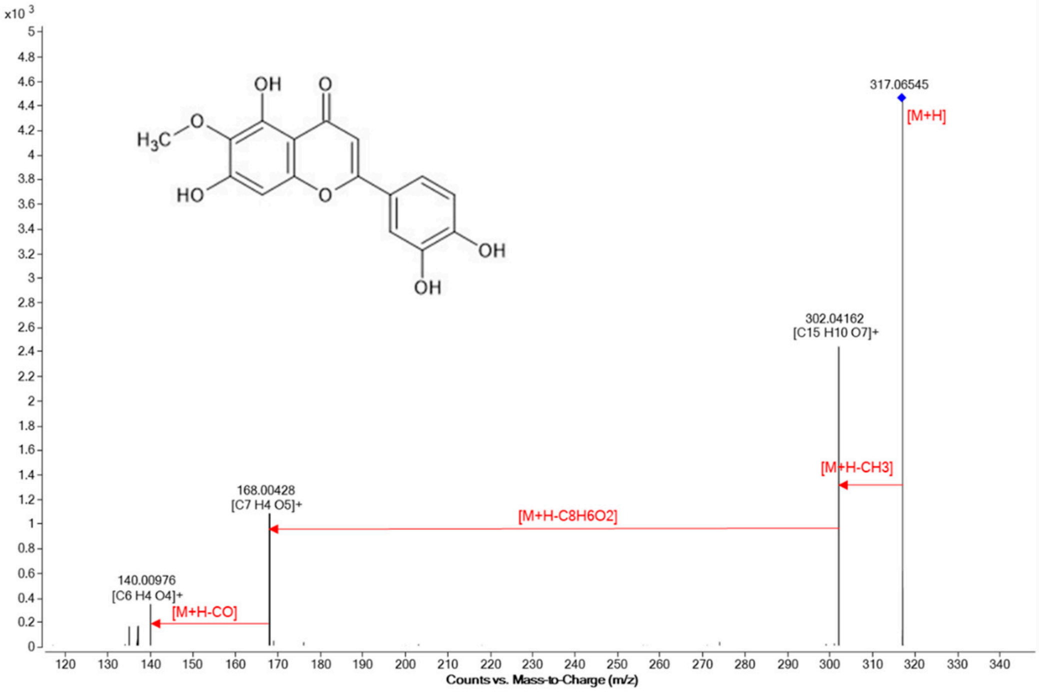

Compound 5
Retention time: 7.443
m/z [M+H]: 315.08646
Formula: $C_{17}H_{14}O_6$
Tentative annotation: Pectolinarigenin
Compound class: Flavone

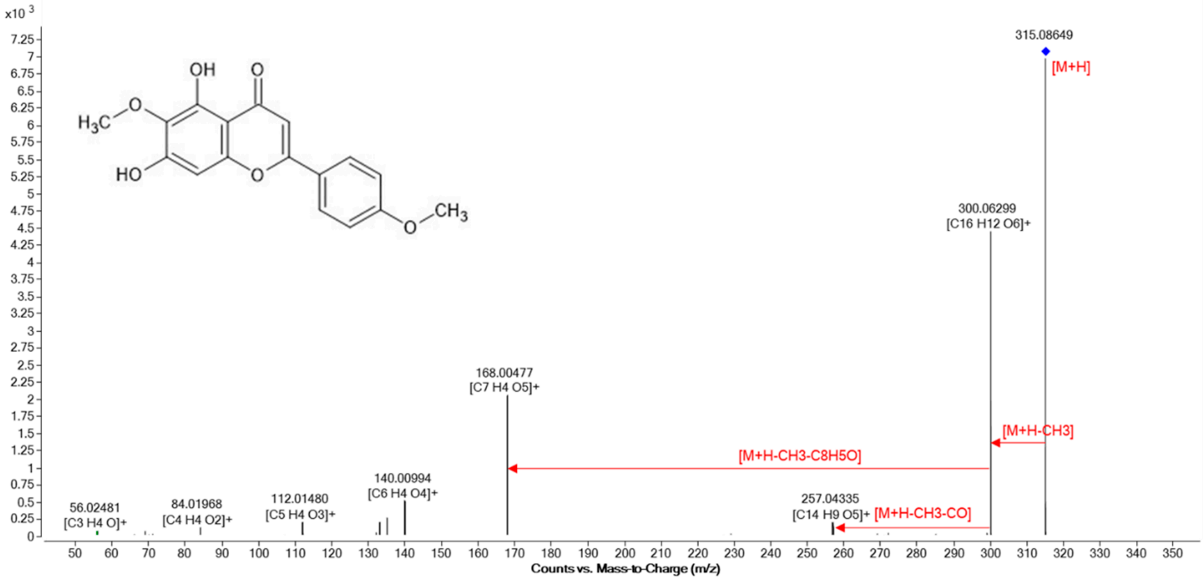

Compound 6
Retention time: 7.578
m/z [M+H]: 301.07016
Formula: $C_{16}H_{12}O_6$
Tentative annotation: Hispidulin
Compound class: Flavone

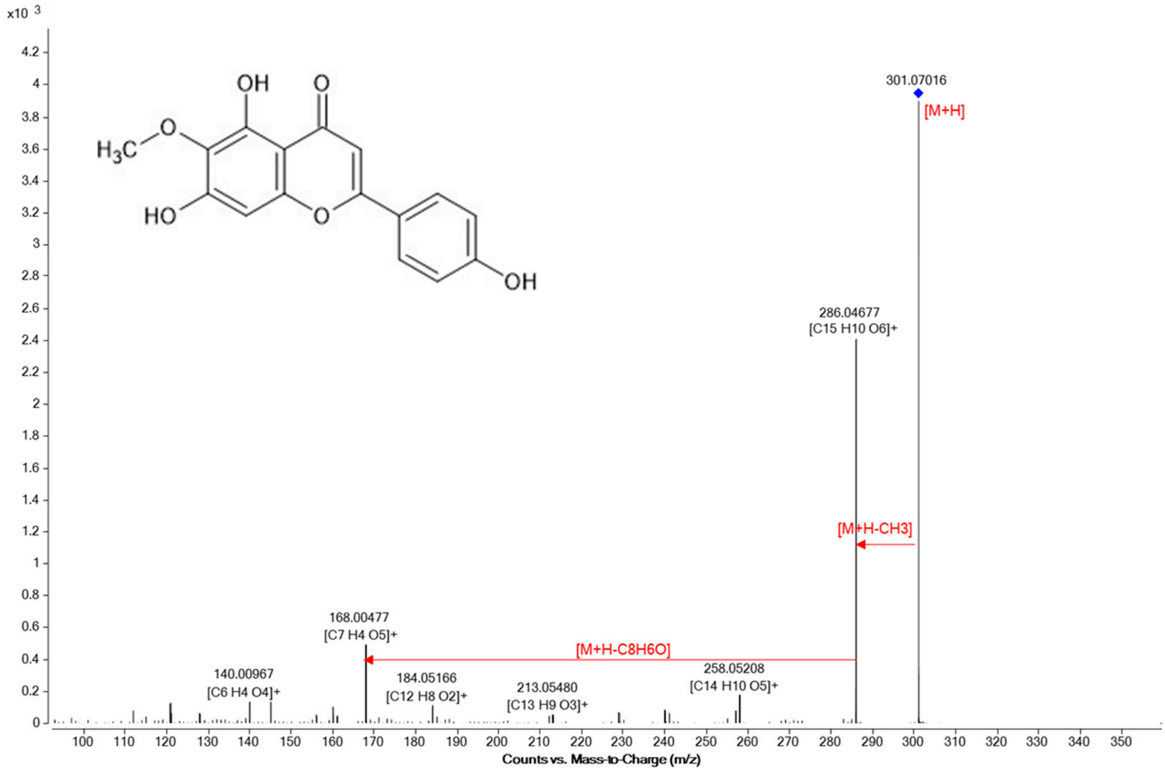

Compound 7
Retention time: 7.631
m/z [M+H]: 271.06018
Formula: $C_{15}H_{10}O_5$
Tentative annotation: Apigenin
Compound class: Flavone

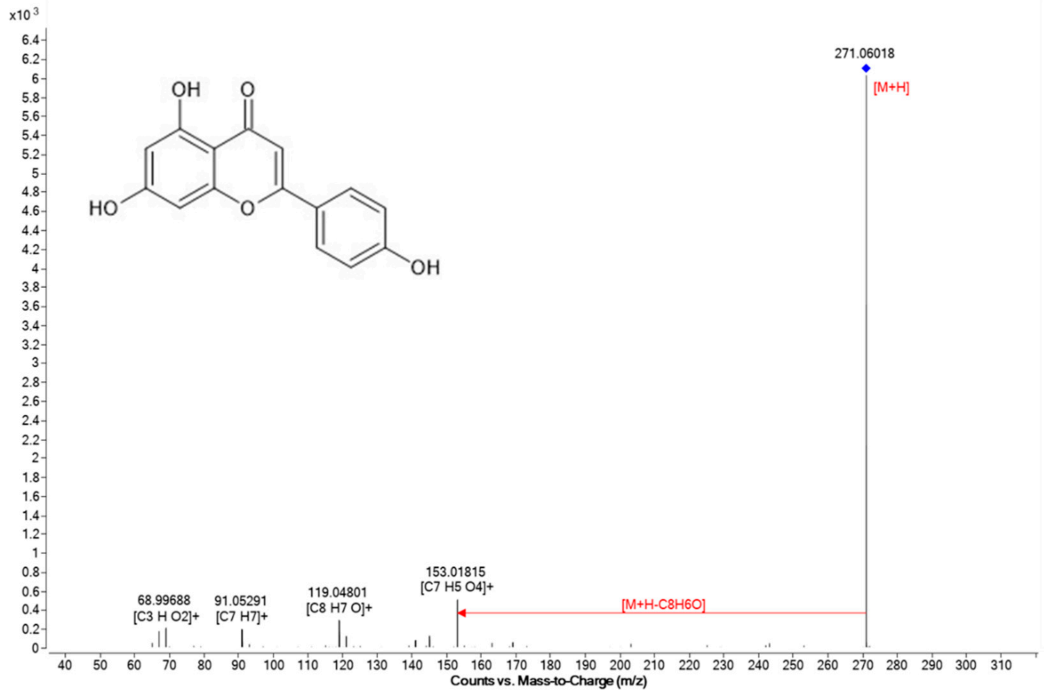

Compound 8
Retention time: 8.254
m/z [M+H]: 301.07061
Formula: $C_{16}H_{12}O_6$
Tentative annotation: 5-O-methylscutellarein
Compound class: Flavone

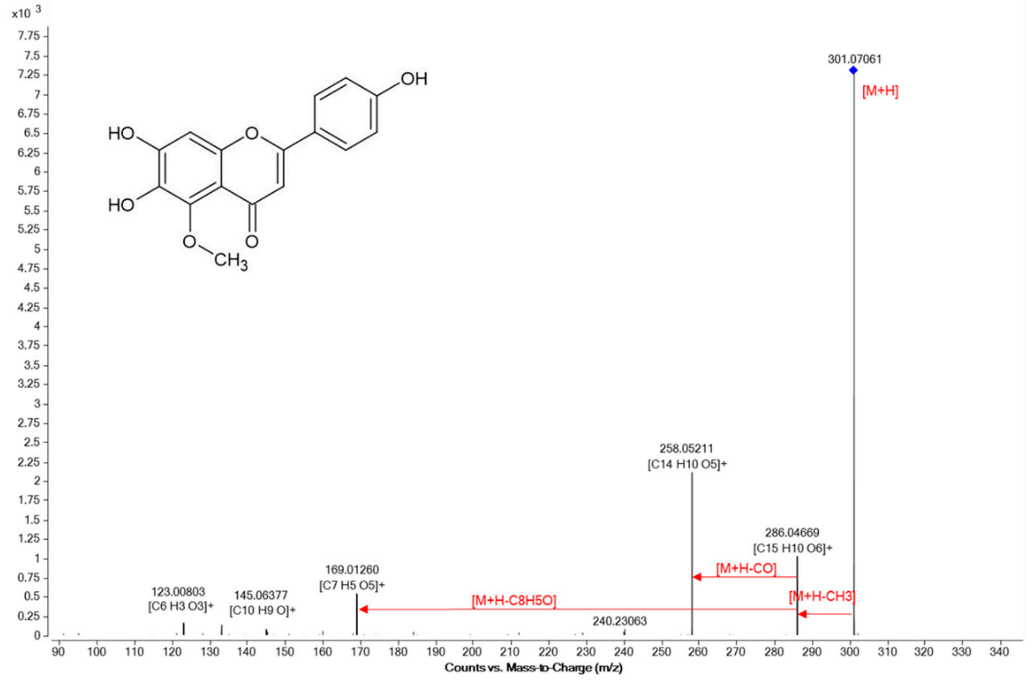

Compound 9
Retention time: 9.331
m/z [M+H]: 285.07564
Formula: $C_{16}H_{12}O_5$
Tentative annotation: Thevetiaflavone
Compound class: Flavone

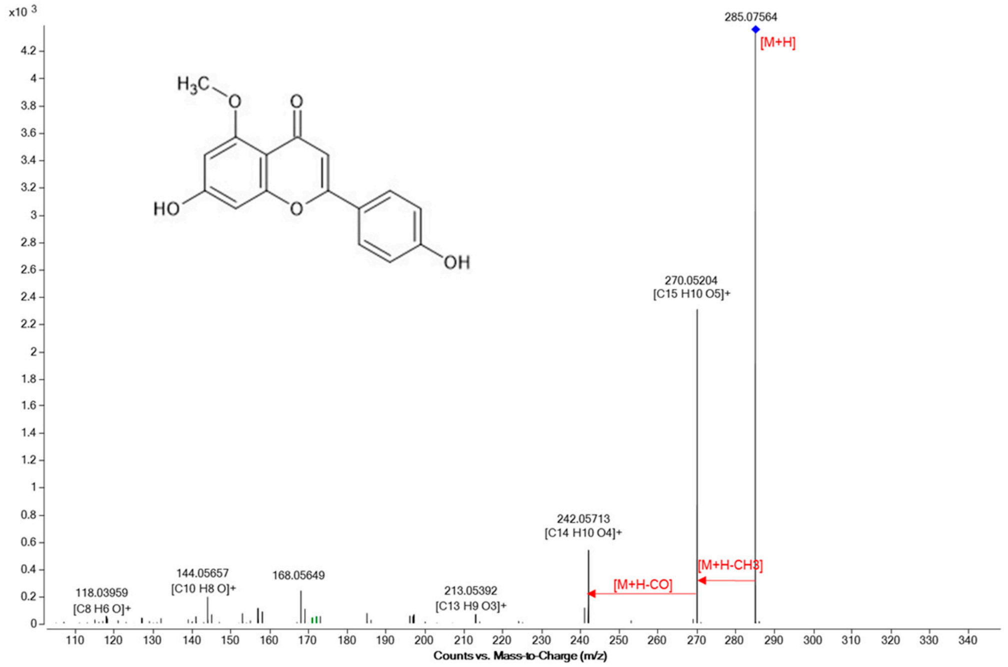

Compound 10
Retention time: 9.572
m/z [M+H]: 285.07554
Formula: $C_{16}H_{12}O_5$
Tentative annotation: Acacetin
Compound class: Flavone

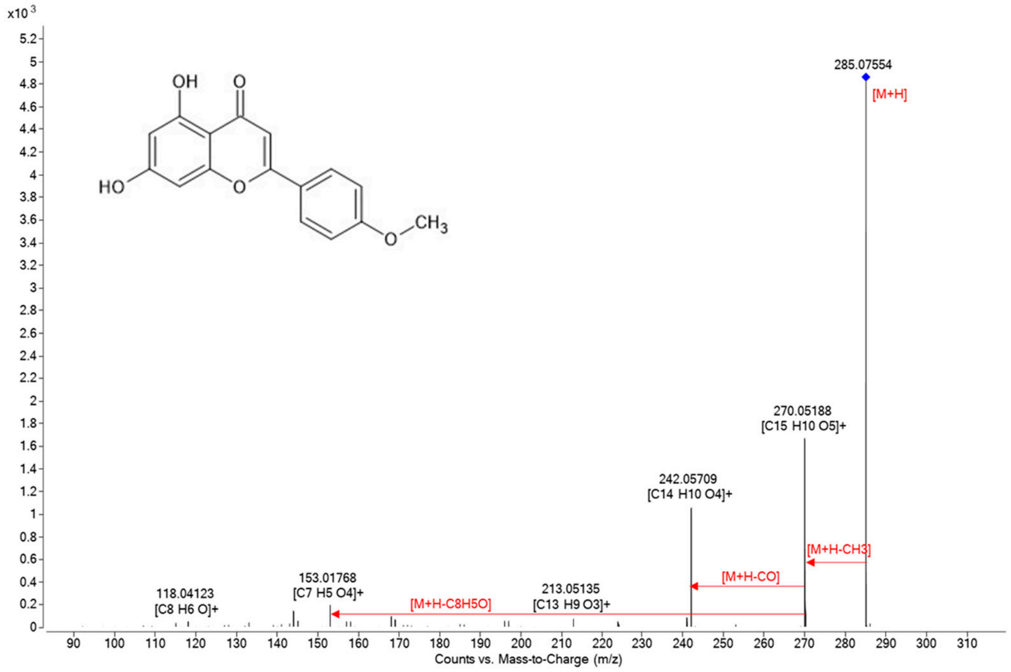

References

1. Behrens, M. D.; Tellis, C. M.; Chagas, M. S. *Arrabidaea chica* (Humb. & Bonpl.) B. Verlot (Bignoniaceae). *Rev. Fitos* **2012**, *7*, 236-244.
2. Ribeiro, A.C.; Telles, T. C.; Ferraz, V. P.; Souza-Fagundes, E. M.; Cassali, G. D.; Carvalho, A. T.; Melo, M. M. Effect of *Arrabidaea chica* extracts on the Ehrlich solid tumor development. *Rev. Bras. Farmacogn*. **2012**, *22*, 364-373.
3. Souza, J. C.; Cordeiro, I.; Lohmann, L.; Ferreira, M. J. P. Flavonoids from *Fridericia speciosa* Mart. (Bignoniaceae). In: 7th Brazilian conference on natural product/ XXXIII resem proceedings **2019**, ISSN: 2594-7346.
4. Takenaka, I. K.; Amorim, J.; Barros, P. A. V.; Brandão, G. C.; Lopes, S. M.; Sales, É. L.; Almeida-Leite, C. M., Martins, F.S.; Cardoso, V. N.; Castilho, R. O.; Fernandes S. O. F. Chemical characterization and anti-inflammatory assessment of the hydroethanolic extract of *Fridericia chica*. *Rev. Bras. Farmacogn*. **2020**, *30*, 559–567.
5. Bojilov, D.; Dagnon, S.; Ivanov, I. New insight into the flavonoid composition of *Chenopodium botrys*. *Phytochem. Lett* **2017**, *20*, 316-321.
6. Cheriet, T.; Ben-Bachir, B.; Thamri, O.; Seghiri, R.; Mancini, I. Isolation and Biological Properties of the Natural Flavonoids Pectolinarin and Pectolinarigenin—A Review. *Antibiotic*s (Basel) **2020**, *9*, 417.
7. Lima, J. C. S.; Godinho, R. O.; Silva, V. C.; Violante, I. M. P.; Macho, A.; Martins, D. T. O. Anti-inflammatory activity of 4′,6,7-trihydroxy-5-methoxyflavone from *Fridericia chica* (Bonpl.) L.G.Lohmann. *Nat. Prod. Res* **2018**, *34*, 726-730.
8. Guinoza, J. T.; Felipe, D. F.; Serra, L. Z.; Junqueira, M.; Aparecida, V.; Cecchini, A.; Ranieri, L. E.; Rodrigues-Filho, E.; Garcia, D. A. Antioxidant Capacity of the leaf extract obtained from *Arrabidaea chica* cultivated in Southern Brazil. *PLoS One* **2013**, *8*, e72733.
